# Near-Infrared Spectroscopy and Continuous Glucose Monitoring During Therapeutic Hypothermia

**DOI:** 10.1089/neur.2023.0053

**Published:** 2024-01-05

**Authors:** Giulia Vagelli, Francesca Garbarino, Maria Grazia Calevo, Giorgia Brigati, Luca Antonio Ramenghi

**Affiliations:** ^1^ Department of Neurosciences, Rehabilitation, Ophthalmology, Genetics, Maternal and Child Health, University of Genoa, Genoa, Italy.; ^2^ Epidemiology and Biostatistics Unit, Neonatal Intensive Care Unit, IRCCS Istituto Giannina Gaslini, Genoa, Italy.; ^3^ Department Mother and Child, Neonatal Intensive Care Unit, IRCCS Istituto Giannina Gaslini, Genoa, Italy.

**Keywords:** continuous glucose monitoring, hypoxic-ischemic encephalopathy, near-infrared spectroscopy, therapeutic hypothermia

## Abstract

The relation between glucose homeostasis and cerebral blood flow (CBF) and their correlation to outcome in neonatal hypoxic-ischemic encephalopathy are unclear. In this short communication, we tried to determine whether changes in regional oxygen saturation (rSO2), as measured by near-infrared spectroscopy (NIRS), in asphyxiated neonates during therapeutic hypothermia correlate with the glycemic profile and whether NIRS and continuous glucose monitoring are useful in identifying cooled asphyxiated neonates at high risk of brain injury. Although there was no correlation between blood glucose and CBF in this small cohort of asphyxiated neonates (13 neonates admitted to the IRCCS Giannina Gaslini NICU in Genoa between March and September 2021), after 24 h of life, increased rSO_2_ and glucose variability with a tendency toward hyperglycemia distinguished neonates who subsequently acquired brain injury from those who did not. As a result of this, it may be possible to monitor cerebral perfusion and metabolic changes as soon as possible after delivery in order to prevent poorer outcomes.

## Introduction

Hypoxic ischemic encephalopathy (HIE) is estimated to affect ∼2–4 per 1000 live births and is the leading cause of death and disability in full-term neonates. Therapeutic hypothermia (TH) has been established as the sole effective therapeutic modality for reducing mortality and improving subsequent neurodevelopmental outcomes.^1,2^ Hypoxic-ischemia (HI) enhances the brain's susceptibility to changes in blood glucose levels, implying that glucose derangements may exacerbate HIE and its unfavorable outcomes. Neonates with HIE are not only at risk for hypoglycemia, but also hyperglycemia. Tam and colleagues^3^ recently discovered that elevated glucose levels on the first day of life were associated with extensive microstructural alterations in the brain during imaging.

Asphyxiated neonates have poor glucose supply to the brain, and experimental studies indicate that glucose may have a direct effect on cerebral blood flow (CBF) regulation,^4^ but the exact nature of the association remains unknown.

The aims of the study were to determine whether changes in regional oxygen saturation (rSO_2_), as measured by near-infrared spectroscopy (NIRS), in asphyxiated neonates during TH correlate with the glycemic profile and whether NIRS and continuous glucose monitoring (CGM) are useful in identifying cooled asphyxiated neonates at high risk of brain injury.

## Methods

We conducted a pilot prospective observational study in which rSO_2_ and CGM were measured concurrently using NIRS in 13 asphyxiated neonates born at or after 35 weeks of gestation who met the criteria for TH and were admitted to the IRCCS Giannina Gaslini NICU in Genoa between March and September 2021. The monitoring period comprised cooling and rewarming for up to 12 h. The rSO_2_ and glycemic patterns in neonates who acquired subsequent brain injury were compared with those who did not at specific time periods (6–24, 24–48, 48–72, and 12 h after rewarming).

## Results

**FIG. 1.**
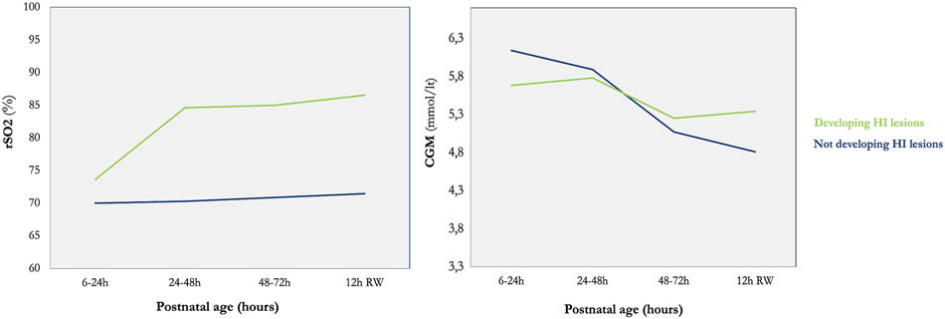
Line charts comparing post-natal rSO_2_ and CGM patterns of the two groups of neonates. There is no correlation between the two variables, but the increasing rSO_2_ in neonates developing HI injury after 24 h of life, as well as the glucose variability and tendency to remain near high values, are clearly shown in comparison to the group of neonates not developing HI injury. rSO_2_, regional oxygen saturation; CGM, continuous glucose monitoring; HI, hypoxic-ischemia.

**FIG. 2.**
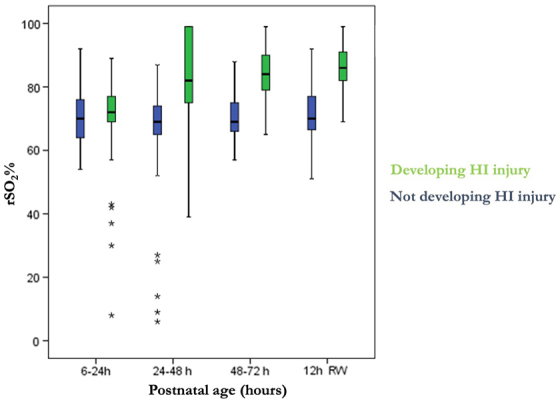
Evolution of regional cerebral oxygenation saturation over the first days of life in asphyxiated neonates treated with hypothermia and 12 h after rewarming comparison based on the development of subsequent brain HI injury using box-and-whisker plots showing median, minimum, and maximum values for each group. Asphyxiated neonates who subsequently acquired brain injury after being treated with hypothermia are labeled as the “developing HI injury” group, whereas neonates who did not acquire such an injury are labeled as the “Not developing HI injury” group. rSO_2_, regional oxygen saturation; HI, hypoxic-ischemia.

There were 11 neonates with available rSO_2_ and CGM data for analysis; 6 acquired brain injury as detected during magnetic resonance imaging (MRI) scans. Glucose derangements had no effect on cerebral hemodynamics regardless of the presence or absence of brain injury (Fig. 1). We detected that neonates who subsequently acquired brain injury had more glycemic derangements with a tendency toward hyperglycemia, compared to neonates who did not acquire brain injury, and that rSO_2_ remained stable in infants with a normal MRI (median, 69%; interquartile range [IQR], 65–75), but significantly increased after 24 h of life onward in infants with brain lesions (median, 81%; IQR, 74–91; Fig. 2).

## Discussion

To the best of our knowledge, this study is the first to concurrently monitor glucose and cerebral oxygenation using CGM and NIRS continuously in asphyxiated neonates for 72 h during TH and 12 h after rewarming.

Although there was no correlation between blood glucose and CBF in this small cohort of asphyxiated neonates, CGM and NIRS monitoring of the glycemic profile and cerebral oxygenation can help predict the risk of acquiring brain injury.

After 24 h of life, increased rSO_2_ and glucose variability with a tendency toward hyperglycemia distinguished neonates who subsequently acquired brain injury from those who did not.

Hyperglycemic episodes in neonates who subsequently acquired brain injury persisted longer than hypoglycemic episodes, implying a complex etiology,^5^ as well as a higher tolerance for intervention in the event of hyperglycemia.

For now, the relationship between blood glucose and cerebral oxygenation during TH and their probable effects on short- and long-term outcome remain unclear and have to be studied in future research. Despite TH, many asphyxiated neonates still die or survive with sequelae of varying degrees, suggesting the necessity for an association with other neuroprotective therapies. As a result of this, it may be possible to monitor cerebral perfusion and metabolic changes as soon as possible after delivery in order to prevent poorer outcomes.

## Abbreviations Used

CBF: cerebral blood flow
CGM: continuous glucose monitoring
HI: hypoxic-ischemia
HIE: hypoxic ischemic encephalopathy
IQR: interquartile range
MRI: magnetic resonance imaging
NIRS: near-infrared spectroscopy
rSO_2_: regional oxygen saturation
TH: therapeutic hypothermia
